# Background Subtraction Based on Three-Dimensional Discrete Wavelet Transform

**DOI:** 10.3390/s16040456

**Published:** 2016-03-30

**Authors:** Guang Han, Jinkuan Wang, Xi Cai

**Affiliations:** College of Information Science and Engineering, Northeastern University, Shenyang 110819, China; coldlight919@163.com (G.H.); wjk@mail.neuq.edu.cn (J.W.)

**Keywords:** background subtraction, three-dimensional discrete wavelet transform, intensity temporal consistency, wavelet shrinkage

## Abstract

Background subtraction without a separate training phase has become a critical task, because a sufficiently long and clean training sequence is usually unavailable, and people generally thirst for immediate detection results from the first frame of a video. Without a training phase, we propose a background subtraction method based on three-dimensional (3D) discrete wavelet transform (DWT). Static backgrounds with few variations along the time axis are characterized by intensity temporal consistency in the 3D space-time domain and, hence, correspond to low-frequency components in the 3D frequency domain. Enlightened by this, we eliminate low-frequency components that correspond to static backgrounds using the 3D DWT in order to extract moving objects. Owing to the multiscale analysis property of the 3D DWT, the elimination of low-frequency components in sub-bands of the 3D DWT is equivalent to performing a pyramidal 3D filter. This 3D filter brings advantages to our method in reserving the inner parts of detected objects and reducing the ringing around object boundaries. Moreover, we make use of wavelet shrinkage to remove disturbance of intensity temporal consistency and introduce an adaptive threshold based on the entropy of the histogram to obtain optimal detection results. Experimental results show that our method works effectively in situations lacking training opportunities and outperforms several popular techniques.

## 1. Introduction

Smart video surveillance systems are extensively applied to various indoor and outdoor scenes nowadays, owing to rapidly increasing demands of security protection, healthcare, home care, *etc.* Moving object detection is a fundamental task of smart video surveillance [1] and has become a hot issue over the last decade [2,3,4,5,6,7,8]. Undoubtedly, background subtraction techniques [9,10,11,12,13,14,15,16,17,18,19] are the most popular in moving object detection.

Traditional background subtraction methods [20,21,22] need a training sequence to build their background models. The training sequence should be sufficiently long and meanwhile clean (without any moving object); however, this is usually hard to satisfy in real-world scenarios, because there are many applications in which clean training data are unavailable or the allowed training time is insufficient.

One example for situations without clean training data is crowded scenes. Continuous moving objects in the crowded scenes (such as airports, train stations, shopping centers and buffet restaurants) make it hard to get clean training data. Researchers have proven that a much longer training phase (up to 800 frames) has to be used to build accurate background models for the crowded scenes [23]. There are also many applications without sufficient training time. One application is short clip analysis. Automatic short clip analysis (such as abstraction, retrieval, *etc.*) is of great value in the current period when smartphone apps for short clips become increasingly popular. Another application is surveillance using a pan-tilt-zoom (PTZ) camera working in intermittent pan mode or preset-position patrol mode. When the PTZ camera changes its field of view and in the meantime a moving object suddenly emerges in the new field of view, the urgent needs for rapid and accurate moving object detection leave no time for training, because key targets often emerge transitorily. Hence, to cope with challenging situations lacking training opportunities that occur frequently in real scenes, background subtraction without a separate training phase becomes a critical task [24].

In this paper, we propose a background subtraction method without any training phase, based on three-dimensional (3D) discrete wavelet transform (DWT). As a transform domain method, we name it TD-3DDWT. TD-3DDWT takes advantage of the frequency domain characteristics of intensity temporal consistency to detect moving objects. A batch of recent gray-scale frames is stored to form a 3D array. Considering that static backgrounds consistent along the time axis correspond to low-frequency components of the 3D array, our method employs the 3D DWT [25] to eliminate those low-frequency components in the wavelet domain. This elimination in the wavelet domain is equivalent to applying a pyramidal 3D filter in the frequency domain, which enables our method to achieve satisfactory results. In addition, we utilize wavelet shrinkage to remove the disturbance of intensity temporal consistency caused by noise, illumination changes, *etc*. Furthermore, we use an adaptive threshold to generate optimal binary detection results. Experimental results prove that our method can rapidly produce accurate detection results in challenging situations lacking training opportunities and outperforms several popular techniques.

The remainder of this paper is organized as follows: We briefly introduce the related work in Section 2. In Section 3, we present our background subtraction method based on 3D DWT. In Section 4, we analyze the merits of our method in detecting both object boundaries and inner parts. Section 5 describes our experimental results and validates the efficiency of our method through comparing its performance with four well-known background subtraction methods. Finally, the conclusion is drawn in Section 6.

## 2. Related Work

A comprehensive review on background subtraction has been provided by Bouwmans [18]. In this section, we introduce recent methods without a separate training phase that are the most related to our work in Section 2.1 and distinguish our method from the other methods based on wavelet transform (WT) in Section 2.2.

### 2.1. Methods without a Separate Training Phase

Visual background extractor (ViBe) [26] utilizes the first frame of a video to quickly initialize its background models and then adaptively adjusts its models through online update. This is a remarkable improvement over methods needing many training frames. All of the improved methods of ViBe [27,28,29] maintain this quick initialization technique, which makes them applicable to short clip analysis. However, for crowded scenes, there is a strong possibility that foreground objects appear in the first frame and cause errors in the initial background models, and it is also hard for the updating mechanism to promptly correct these errors owing to persistent foreground objects in the following frames. This will lead to many ghosts (a ghost is a set of connected points, which are detected as motion, but not corresponding to any real moving object) in the detection results of crowded scenes, especially for short clips.

Robust principal component analysis (RPCA) algorithms [30,31,32,33,34] have been proven successful at separating foreground objects from the background without a training sequence. They assume that the underlying background images are linearly correlated, and the matrix composed of vectorized video frames can be decomposed into a low-rank matrix (static background) and a sparse matrix (foreground objects). RPCA methods can directly extract foreground objects from a batch of consecutive frames. However, their application is limited, because the optimization algorithms used for matrix decomposition (such as principal component pursuit and outlier pursuit) suffer from two pitfalls: performance degradation with dimension increase and computational intractability.

Tsai and Chiu [35] have presented a background subtraction method using two-dimensional (2D) discrete Fourier transform (DFT). Since it works in the transform domain, we name it TD-2DDFT. TD-2DDFT converts input frames to gradient images, applies the 2D DFT on each spatial-temporal slice of the gradient image sequence and removes the vertical line pattern of the static backgrounds. In this way, TD-2DDFT can detect foreground objects without a training phase. However, its detection results contain incomplete object information (only boundaries of moving objects) and also have ringing around object boundaries.

### 2.2. Methods Based on WT

So far, all of the previous works based on WT, in essence, use the same well-established moving object detection framework in which foreground objects are detected according to the differences of features between adjacent frames or between the current frame and background models. The previous works only employ various two-dimensional (2D) WTs to extract approximate coefficients and wavelet coefficients as features, in order to calculate differences between adjacent frames [36,37,38,39] or between the current frame and background models [40,41,42,43,44].

However, the framework of our method is completely different from the abovementioned framework of the other works. After analyzing frequency domain characteristics of the intensity temporal consistency of static backgrounds, we introduce three-dimensional (3D) DWT to decompose the data cube (constructed by a batch of consecutive frames) into multiple 3D DWT sub-bands and establish the relationship between static backgrounds and certain 3D DWT sub-bands. In this way, the background can be separated from the foreground in different 3D DWT sub-bands. Therefore, by discarding sub-bands corresponding to the backgrounds, our method can directly remove the background and retain the foreground.

## 3. Background Subtraction Based on 3D DWT

### 3.1. Analysis of Background Removal in the 3D Wavelet Domain

In our work, we remove static backgrounds by using the frequency domain characteristics of intensity temporal consistency. In this subsection, we further detail the rationality of our method to remove the static backgrounds in the 3D wavelet domain by taking advantage of multiscale analysis characteristics of DWT.

**Figure 1:**
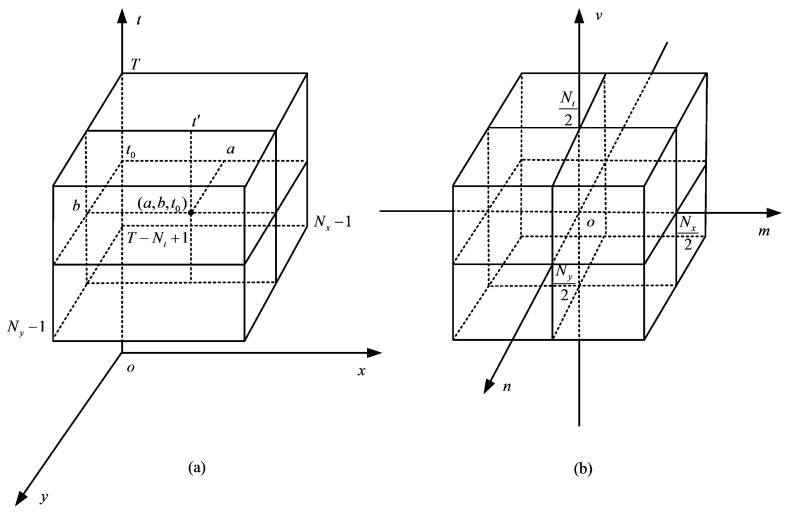
Illustration of a three-dimensional (3D) array f(x,y,t) and its centralized 3D frequency space F(m,n,v) : (**a**) f(x,y,t); (**b**) F(m,n,v).

For the convenience of analysis, we first let $N_{x}\times N_{y}$ denote the size of the input frames and then construct a 3D array f(x,y,t) of size $N_{x}\times N_{y}\times N_{t}$ from a batch of $N_{t}$ consecutive gray-scale frames. In a 3D coordinate system in which *x*, *y* and *t* represent row, column and time, respectively, a scheme of f(x,y,t) is shown in Figure 1a, for $x=0,1,...,N_{x}-1$; $y=0,1,...,N_{y}-1$ and $t=T-N_{t}+1,T-N_{t}+2,...,T$, where *T* denotes the current time. At a certain moment $t_{0}$, $f(x,y,t_{0})$ is a 2D spatial image. We draw a line $t^{\prime }$, parallel to the *t* axis, passing through an arbitrary point (*a*,*b*,*t*_0_). It can be expected that the stored intensities along the line $t^{\prime }$ will be approximately the same, if the location (*a*,*b*) in the spatial domain is part of a static background over the entire $N_{t}$ observed frames. This phenomenon is called intensity temporal consistency.

Static backgrounds characterized by intensity temporal consistency in the 3D space-time domain correspond to low-frequency information in the 3D frequency domain. In other words, static backgrounds form the low-frequency components along the *v* axis in Figure 1b. Figure 1b shows the centralized 3D frequency space F(m,n,v), where *m*, *n* and *v* are the frequency domain variables that correspond to *x*, *y* and *t*, respectively. To remove the static backgrounds, we need to introduce high pass filters along the *v* axis.

**Figure 2:**
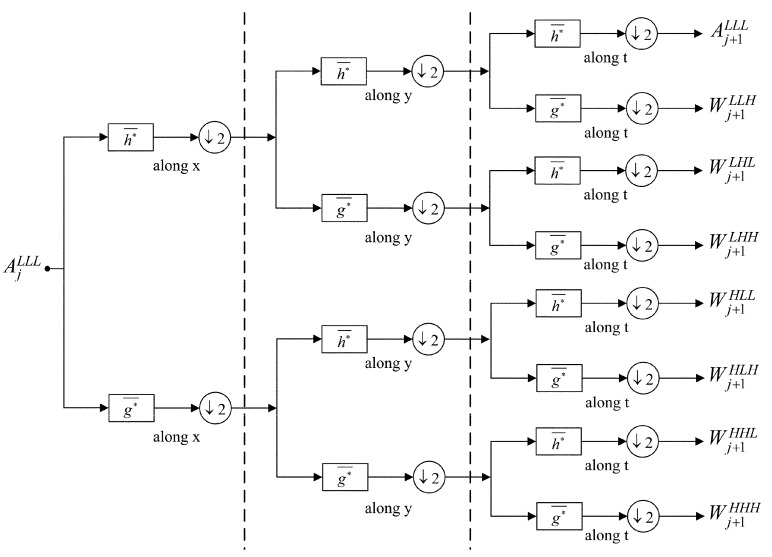
Block diagram of the analysis filter bank of the 3D discrete wavelet transform (DWT).

Rather than directly implementing high pass filtering in the frequent domain, we introduce the 3D DWT [25] to perform the high pass filtering in the wavelet domain by making use of its multiscale analysis. Figure 2 shows the block diagram of the analysis filter bank of the 3D DWT, in which scaling vector $\bar{h^{*}}$ is used as a low pass filter; wavelet vector $\bar{g^{*}}$ is used as a high pass filter [45]; sub-band $A_{j}^{LLL}$ defines an approximation of f(x,y,t) at scale *j* (1≤j≤J), and $A_{0}^{LLL}=f(x,y,t)$. After decomposition, $A_{j}^{LLL}$ is decomposed into eight sub-bands (*i.e.*, an approximation sub-band $A_{j+1}^{LLL}$ and seven detail sub-bands $W_{j+1}^{LLH}$, $W_{j+1}^{LHL}$, $W_{j+1}^{LHH}$, $W_{j+1}^{HLL}$, $W_{j+1}^{HLH}$, $W_{j+1}^{HHL}$, $W_{j+1}^{HHH}$) at scale *j* + 1, and further decomposition can be implemented on the sub-band $A_{j+1}^{LLL}$ in a similar manner. Here, *L* denotes the low-frequency components, and *H* denotes the high-frequency components. For each scale *j* (1≤j≤J), the sub-bands $A_{j}^{LLL}$, $W_{j}^{LHL}$, $W_{j}^{HLL}$ and $W_{j}^{HHL}$ contain all of the low-frequency components along the *t* axis (that is, the low-frequency information along the *v* axis in the 3D frequency domain).

Since multiscale analysis enables us to decompose the 3D array f(x,y,t) into different frequency bands, we can modify resulting sub-band coefficients at each scale to eliminate undesired low-frequency components along the *v* axis. In this way, we can remove the static backgrounds while reserving the foreground objects.

### 3.2. Procedure of TD-3DDWT

#### 3.2.1. Static Backgrounds Removal

After loading a batch of consecutive input frames, we convert color images to gray-scale images and then construct a 3D array f(x,y,t) from these gray-scale images. To remove static backgrounds, we decompose f(x,y,t) into *J* levels using the 3D DWT, then set the coarsest approximation (*i.e.*, the sub-band $A_{J}^{LLL}$) to zero and meanwhile set the detail sub-bands $W_{j}^{LHL}$, $W_{j}^{HLL}$, $W_{j}^{HHL}$ at each scale *j* (1≤j≤J) to zero, namely:

(1) $$A_{J}^{LLL}=0$$

(2) $$W_{j}^{LHL}=W_{j}^{HLL}=W_{j}^{HHL}=0\;(1\leq j\leq J)$$

In this way, the low-frequency components along the *v* axis (corresponding to the static backgrounds) are removed in the 3D wavelet domain.

#### 3.2.2. Disturbance Removal

Disturbance (such as noise and illumination changes) will pose a threat to the intensity temporal consistency of static backgrounds and, hence, should be eliminated to reduce its influence on our detection results. Considering that disturbance corresponds to small wavelet coefficients and foreground objects usually correspond to large wavelet coefficients, we employ the wavelet shrinkage [46] to remove the disturbance. The modified wavelet coefficients are defined as follows:

(3) $${\hat{W}}_{j,k}^{D}=\{\begin{array}{ll} \mathrm{sgn}\left(W_{j,k}^{D}\right)\times \left(\left|W_{j,k}^{D}\right|-\lambda_{j}^{D}\right) & \left|W_{j,k}^{D}\right|\geq \lambda_{j}^{D}\\ 0 & \left|W_{j,k}^{D}\right|<\lambda_{j}^{D} \end{array}$$

where $W_{j,k}^{D}$ indicates the *k*-th wavelet coefficient in the detail sub-band $W_{j}^{D}$ (*D* = *LLH*, *LHH*, *HLH*, *HHH*), $\lambda_{j}^{D}$ denotes the threshold for $W_{j}^{D}$ and ${\hat{W}}_{j,k}^{D}$ signifies the modified *k*-th wavelet coefficient.

The threshold $\lambda_{j}^{D}$ here is defined as:

(4) $$\lambda_{j}^{D}={\hat{\sigma }}_{j}^{D}\times \sqrt{2\log\left(N_{x}\times N_{y}\times N_{t}\right)}$$

where ${\hat{\sigma }}_{j}^{D}$ is the estimation of the disturbance level in the detail sub-band $W_{j}^{D}$ and can be computed as:

(5) $${\hat{\sigma }}_{j}^{D}=\frac{MAD}{0.6745}$$

where *MAD* is the median absolute deviation of the wavelet coefficients at the finest level *j* = 1.

#### 3.2.3. Detection Results Generation

After the removal of static backgrounds and disturbance, a 3D array g(x,y,t) is reconstructed from the modified sub-band coefficients by using 3D inverse DWT. Then, we employ thresholding to obtain binary results from reconstructed frames. Since a fixed empirical threshold is usually unable to fit all frames, we employ an adaptive threshold instead. The adaptive threshold is based on the entropy of the histogram [47] and is used here to find the optimal threshold for each reconstructed frame. Final detection results can be achieved by comparing each frame in g(x,y,t) with the frame-level adaptive threshold.

## 4. Merits of TD-3DDWT

**Figure 3:**
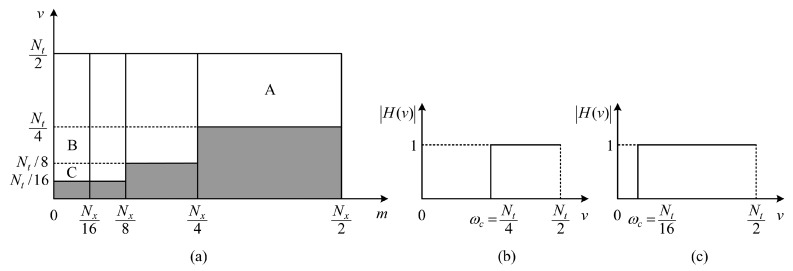
Analysis of our pyramidal 3D filter utilizing the three-scale 3D DWT: (**a**) Scheme of a m−v slice, where white parts denote support regions and gray parts denote non-support regions; (**b**) one-dimensional (1D) ideal high pass filters (IHPFs) along the *v* axis when $N_{x}/4<m<N_{x}/2$; (**c**) 1D IHPFs along the *v* axis when $0<m<N_{x}/16$.

Owing to the characteristics of multiscale analysis, the processing we carry out to remove static backgrounds in the wavelet domain is essentially equivalent to utilizing a pyramidal 3D filter in the frequency domain, and this filter is comprised of many one-dimensional (1D) ideal high pass filters (IHPFs) along the *v* axis with different cutoff frequencies (*i.e.*, $\omega_{c}$) in different frequency bands of the *m* and *n* axes. For the convenience of description, we take a cross-sectional slice of the preceding pyramidal 3D filter through the origin orthogonal to the *m* axis or the *n* axis. For instance, Figure 3a shows a m−v slice of our pyramidal 3D filter utilizing the three-scale 3D DWT (since the frequency spectrum is symmetrical, we only show the first quadrant). As depicted in Figure 3a, white parts denote support regions where the wavelet coefficients are reserved, and gray parts denote non-support regions where the wavelet coefficients are zeroed. The horizontal borders (*i.e.*, $v=N_{t}/4$, $v=N_{t}/8$ and $v=N_{t}/16$) between the white parts and the gray parts signify cutoff frequencies of the 1D IHPFs along the *v* axis.

Roughly, along the *v* axis (corresponding to the *t* axis in Figure 1a), the low-frequency components indicate information about the static backgrounds, and the high-frequency components indicate information about the moving objects; along the *m* axis (corresponding to the *x* axis in Figure 1a), the low-frequency components signify information about the smooth areas, and the high-frequency components signify information about the edges. Consequently, in Figure 3a, Support Region A likely corresponds to the edges of moving objects; Support Regions B and C likely correspond to the inner smooth areas of moving objects.

Figure 3b,c show the amplitude-frequency characteristics of 1D IHPFs along the *v* axis when $N_{x}/4<m<N_{x}/2$ and $0<m<N_{x}/16$, respectively. As illustrated in Figure 3b, in high-frequency bands of the *m* axis, the cutoff frequencies of 1D IHPFs along the *v* axis are larger (*i.e.*, $\omega_{c}=N_{t}/4$); Figure 3c shows that in low-frequency bands of the *m* axis, the cutoff frequencies of 1D IHPFs along the *v* axis are smaller (*i.e.*, $\omega_{c}=N_{t}/16$). The 1D IHPFs along the *v* axis in our method have different cutoff frequencies, which are determined by the sub-bands of the multiscale 3D DWT.

This characteristic brings two advantages to TD-3DDWT:

1. Our method weakens the ringing on the object boundaries and, thus, locates the object boundaries precisely. As we know, the ringing behavior is a characteristic of IHPFs, and the cutoff frequency affects the range of the ringing. By increasing the cutoff frequency, the ringing will be reduced. In Figure 3a, when $N_{x}/4<m<N_{x}/2$, since the 1D IHPFs along the *v* axis have larger cutoff frequencies (*i.e.*, $\omega_{c}=N_{t}/4$ shown in Figure 3b), the ringing around the edges of moving objects (corresponding to the Support Region A) is slight and imperceptible.
2. The detection results of our method include not only the object boundaries, but also the inner parts of moving objects. In Figure 3a, the Support Regions B and C correspond to smooth areas inside the moving objects, and they can be reserved in our method, so that the detection results of our method can include the inner parts of moving objects. Although the 1D IHPFs along the *v* axis are with smaller cutoff frequencies (*i.e.*, $\omega_{c}=N_{t}/16$ in Figure 3c) when $0<m<N_{x}/16$, the ringing mainly emerges inside the moving objects and, hence, does not affect the detection of object boundaries.

## 5. Experimental Results

In this section, we first introduce our experimental setup in Section 5.1, including the test sequences used in our experiments, the optimal values for the parameters of our method and four well-established methods used for comparison. Then, we provide the results of our method and compare them with those of the other methods in terms of qualitative and quantitative evaluations in Section 5.2 and Section 5.3, respectively.

### 5.1. Experimental Setup

#### 5.1.1. Test Sequences

To prove the ability of our method in background subtraction without a separate training phase, we mainly focus on the performance of detection methods on situations lacking training opportunities, including situations without sufficient training time (e.g., short clips and PTZ camera sequences) and situations without clean training data (e.g., crowded scenes with continuous moving objects).

To be specific, in the experiments, we first use the short clips and PTZ camera sequences to test the ability of each method in dealing with situations without sufficient training time; we then use the long crowded sequences to verify the capability of each method in coping with situations without clean training data.

At first, we select six crowded test sequences, each of which has no clean training data because of moving objects in its initial frames and the continuous flow of moving objects throughout the sequence. To be specific, we select three typical crowded indoor scenarios from the I2R dataset [48] (including a shopping center, an airport and a buffet restaurant) and three typical crowded outdoor sequences from the ChangeDetection.net (CDnet) benchmark dataset [49] (including a skating sequence in the bad weather category, a tram crossroad sequence and a turnpike sequence in the low framerate category). For each selected indoor sequence coming from the I2R dataset, twenty manually-labeled ground-truth references are provided in [50]; for each outdoor sequence of the CDnet benchmark dataset, all of the test data (after hundreds of training frames) are provided with manually-labeled ground-truth references [51].

From each of the abovementioned selected test sequences, we cut a short clip of 96 frames (shorter than a typical training phase of a traditional background substation method). In each short clip, many foreground objects are appearing in the scene, especially in the first frame. In this way, we get three indoor short clips, including short clip SC96 (Frames 1820 to 1915 from the shopping center sequence), short clip AP96 (Frames 3400 to 3495 from the airport sequence) and short clip BR96 (Frames 1550 to 1645 from the buffet restaurant sequence); and we also have three outdoor short clips, including short clip SK96 (Frames 1905 to 2000 from the skating sequence), short clip TC96 (Frames 413 to 508 from the tram crossroad sequence) and short clip TP96 (Frames 800 to 895 from the turnpike sequence). For each indoor short clip, within its first ten frames, there is one frame with a ground-truth reference; for each outdoor short clip, all of the ground-truth references for the 96 frames are available.

Besides, we also select two PTZ camera sequences (including an intermittent pan sequence and a two-position PTZ camera sequence) from the PTZ category of CDnet. For a panning PTZ camera, when it stops to rest on one of its preset positions, an urgent demand for rapid and accurate detection of moving objects in the camera’s new field of view leaves no time for training, because the moving objects in the new field of view may be key targets emerging transitorily. For the intermittent pan sequence, it is captured by a PTZ camera that pans intermittently from one preset position to the other preset position, and the interval between two consecutive panning movements is about 16 frames. For the two-position PTZ camera sequence, it is recorded by a PTZ camera working in two-position patrol mode in which the PTZ camera continuously pans with a high speed from one preset position to the other preset position. Furthermore, for either of the PTZ camera sequences, all of the test data (except for the frames captured when the PTZ camera is panning) are provided with manually-labeled ground-truth references.

Moreover, to test the performance of our method in general environments, we also use the baseline category of the CDnet containing four videos (*i.e.*, highway, office, pedestrians and PETS2006) to show the efficiency of our method for normal videos.

#### 5.1.2. Analysis and Determination of Our Parameters

In our method, there are three parameters: wavelet filter *wfilter*, decomposition scale *J* and number of frames in each batch $N_{t}$.

**Figure 4:**
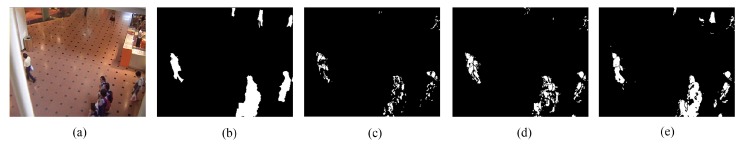
Our detection results of Frame 1780 of the shopping center sequence for *J* ranging from two to four: (**a**) input frame; (**b**) ground-truth reference; (**c**) result when *J* = 2; (**d**) result when *J* = 3; (**e**) result when *J* = 4.

**Figure 5:**
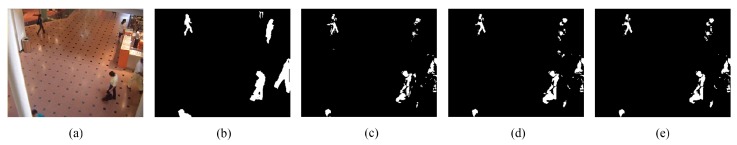
Our detection results of Frame 1740 of the shopping center sequence when $N_{t}$ equals 32, 64 and 128, respectively: (**a**) input frame; (**b**) ground-truth reference; (**c**) result when $N_{t}$ equals 32; (**d**) result when $N_{t}$ equals 64; (**e**) result when $N_{t}$ equals 128.

- ***Wavelet filter*:** In most applications, the wavelet filters with the support width ranging from five to nine are appropriate. The wavelet filter with a larger support width will result in a border problem, and the wavelet filter with a smaller support width is disadvantageous for concentrating the signal energy. In our experiments, we find that the choice of the *wfilter* does not affect our results very much. Consequently, we empirically set the *wfilter* = db5 throughout our test.
- ***Decomposition scale*:** To ensure effective decomposition, the decomposition scale *J* should satisfy the condition that $2^{J}<N_{t}$. As mentioned before, in order to detect smooth areas inside the moving objects, we need to reserve the low-frequency components in support regions, such as B and C in Figure 3a. Hence, in the permitted range, *J* should be as large as possible. To testify to our analysis, we fix other parameters as $N_{t}=32$ and *wfilter* = db5, while performing our method on the shopping center sequence, and obtain detection results illustrated in Figure 4 for *J* ranging from two to four. According to Figure 4, it is evident that the result is better for a larger *J*, and we also find that, to gain satisfactory results, *J* should be no smaller than 4, *i.e.*, J≥4.
- ***Number of frames in each batch*:** Given a decomposition scale *J*, the number of frames in each batch $N_{t}$ should satisfy the condition that $N_{t}>2^{J}$. Moreover, to get rid of the border problem, we should further set $N_{t}\geq 2^{J+1}$. Here, we suppose *J* = 4 and set *wfilter* = db5. To determine an optimal value for $N_{t}$, we visually compare the results shown in Figure 5 with different $N_{t}$. As can be seen, no significant improvements are achieved as the $N_{t}$ increases. However, the memory cost is indeed increasing when we set a larger $N_{t}$. Therefore, we should set the $N_{t}$ as small as possible in its permitted range.

In conclusion, throughout the test, three parameters of our method are set as follows: the wavelet filter *wfilter* = db5, the decomposition scale *J* = 4 and the number of frames in each batch $N_{t}=32$.

#### 5.1.3. Methods Considered for the Comparison and Their Parameter Settings

For the comparison, we introduce four well-known algorithms listed below:

- ViBe is a background subtraction algorithm using a quick initialization technique realized by random sampling in the first frame. We set the parameters exactly the same as Barnich and Van Droogenbroeck [26], namely the number of background samples *N* = 20, the distance threshold *R* = 20, the minimal cardinality ${\#}_{\min}=2$ and the time subsampling factor ϕ=16.
- PCP (principal component pursuit) is the state-of-the-art algorithm for RPCA. There are two main parameters in PCP: number of frames in each batch $N_{t}$ and regularization parameter λ. For the permission of memory cost and also for fair comparison with our method, we set $N_{t}=32$ in our experiments. For λ, we choose exactly the same as Candès *et al.* [31], namely $\lambda =1/\sqrt{\max(n_{1},n_{2})}$, where $n_{1}$ and $n_{2}$ are the row and column dimensions of the input frames, respectively.
- TD-2DDFT is a transform domain method using the 2D DFT. The parameters are also set as Tsai and Chiu [35], namely number of frames in each batch $N_{t}=11$ and notch width of filter Δw=3.
- Crnojević *et al.* [36] proposed a transform domain method using 2D undecimated wavelet transform (UWT) (TD-2DUWT). TD-2DUWT is based on the framework of frame differencing, and, hence, does not need a separate training phase. In our experiments, we use exactly the three-scale 2D Haar UWT as in [36].

#### 5.1.4. Other Settings

For a fair comparison, no post-processing techniques (noise filtering, morphological operations, connected components analysis, *etc.*) are applied in our test with the purpose to evaluate the unaided strength of each approach.

### 5.2. Visual Comparisons

We provide the detection results of the six short clips and two PTZ camera sequences by using the five methods for visual comparisons in Figure 6, Figure 7, Figure 8, Figure 9, Figure 10, Figure 11, Figure 12 and Figure 13, respectively.

**Figure 6:**
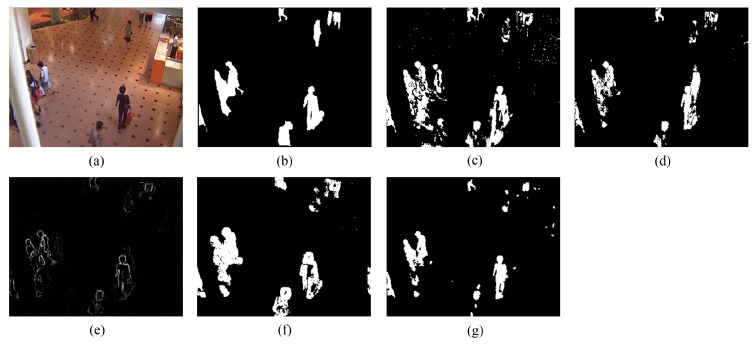
Detection results of the eighth frame of SC96 (Frame 1827): (**a**) input frame; (**b**) ground-truth reference; (**c**) result of the visual background extractor (ViBe); (**d**) result of principal component pursuit (PCP); (**e**) result of the transform domain method based on 2D DFT (TD-2DDFT); (**f**) result of the transform domain method based on 2D undecimated wavelet transform (UWT) (TD-2DUWT); (**g**) result of the transform domain method based on 3D DWT (TD-3DDWT).

Figure 6 exhibits the detection results of the eighth frame of SC96 (Frame 1827 with the manually-labeled ground-truth reference). As depicted in Figure 6c, ViBe produces a polluted result (containing plenty of ghosts). This is because foreground objects in the first frame of SC96 lead to errors in ViBe’s initial background models, and constant foreground objects in the following frames make it difficult to quickly converge to accurate background models, even if ViBe introduces an excellent update mechanism to correct errors after quick initialization. PCP yields an acceptable result, but significant shadows are detected along with the person carrying an orange bag. TD-2DDFT yields a result including only incomplete object boundaries with moderate ringing around, for the inputs of TD-2DDFT are gradient images. TD-2DUWT produces a result with much “fatter” foreground objects. This is because the 2D UWT up-samples the impulse responses of its filter bank [52], which makes the foreground objects expand their boundaries in wavelet sub-bands after multi-scale decomposition and then affects final foreground localization. In contrast, Figure 6g shows that the result of our method contains relative complete moving objects with no ghosts and few shadows. 

**Figure 7:**
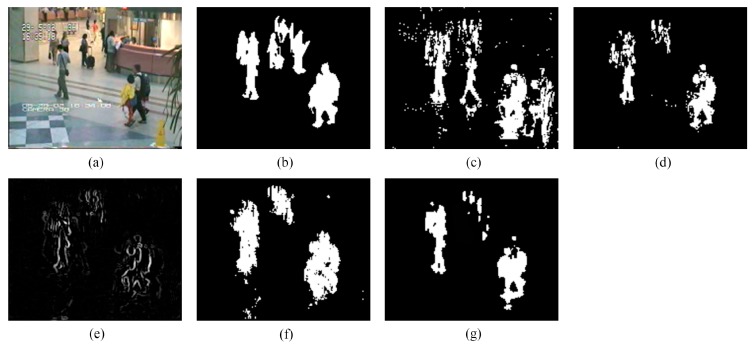
Detection results of the 10th frame of AP96 (Frame 3409): (**a**) input frame; (**b**) ground-truth reference; (**c**) result of ViBe; (**d**) result of PCP; (**e**) result of TD-2DDFT; (**f**) result of TD-2DUWT; (**g**) result of TD-3DDWT.

**Figure 8:**
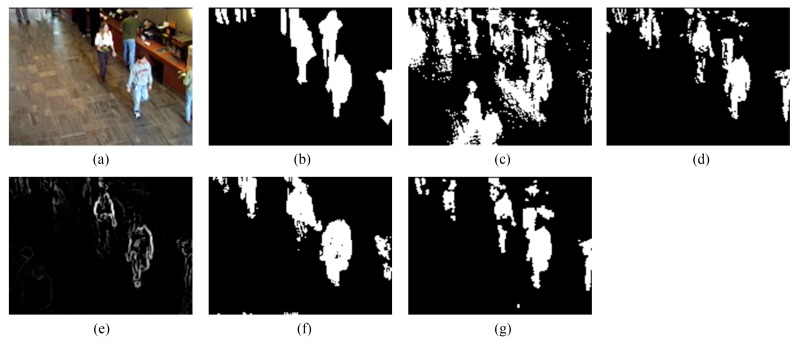
Detection results of the ninth frame of BR96 (frame 1558): (**a**) input frame; (**b**) ground-truth reference; (**c**) result of ViBe; (**d**) result of PCP; (**e**) result of TD-2DDFT; (**f**) result of TD-2DUWT; (**g**) result of TD-3DDWT.

As can be seen from Figure 7 and Figure 8, our method still achieves the best results. However, in Figure 7g, a person standing still with a suitcase is not detected; in Figure 8g, shadows in the top left are detected. This is because our method utilizes intensity temporal consistency and, hence, is unable to cope with still foreground objects and significant shadows. On the other hand, owing to the intensity temporal consistency we employed, for other challenges (such as sudden illumination changes, moved background objects, inserted background objects and beginning moving objects), our method can immediately capture new intensity temporal consistency in the next batch and promptly recover from false detections.

**Figure 9:**
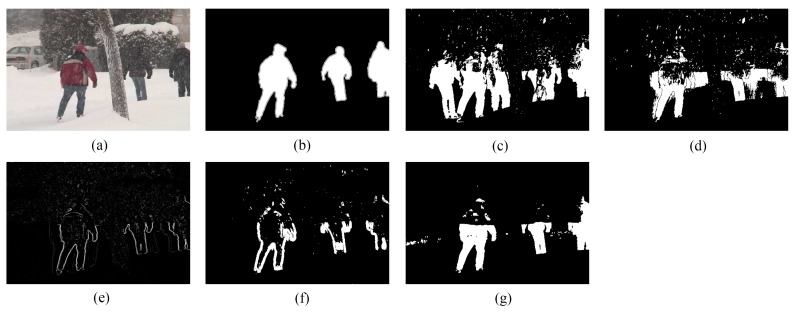
Detection results of the 49th frame of SK96 (Frame 1953): (**a**) input frame; (**b**) ground-truth reference; (**c**) result of ViBe; (**d**) result of PCP; (**e**) result of TD-2DDFT; (**f**) result of TD-2DUWT; (**g**) result of TD-3DDWT.

**Figure 10:**
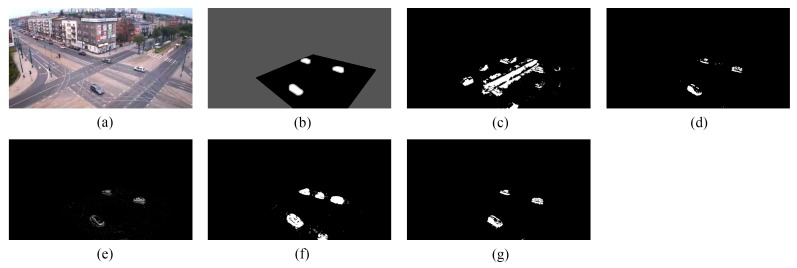
Detection results of the 90th frame of TC96 (Frame 502): (**a**) input frame; (**b**) ground-truth reference; (**c**) result of ViBe; (**d**) result of PCP; (**e**) result of TD-2DDFT; (**f**) result of TD-2DUWT; (**g**) result of TD-3DDWT.

**Figure 11:**
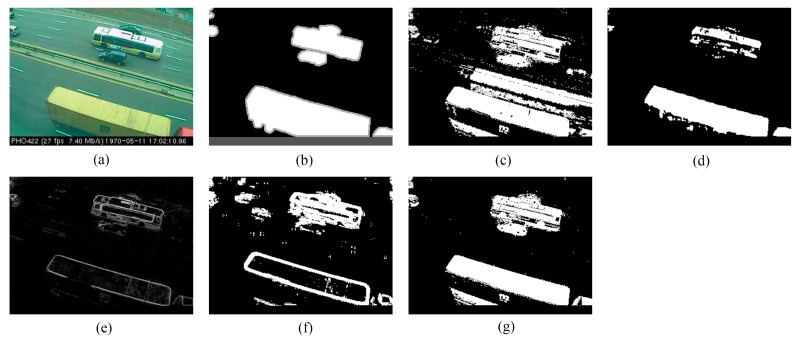
Detection results of the 89th frame of TP96 (Frame 888): (**a**) input frame; (**b**) ground-truth reference; (**c**) result of ViBe; (**d**) result of PCP; (**e**) result of TD-2DDFT; (**f**) result of TD-2DUWT; (**g**) result of TD-3DDWT.

Figure 9, Figure 10 and Figure 11 show the detection results of the three outdoor short clips. It is worth mentioning that ViBe suffers from severe ghosts throughout the short clips, no matter in the middle (e.g., Figure 9c) or at the end (e.g., Figure 10c and Figure 11c) of a short clip; and TD-2DUWT is bothered with “holes” inside the detected objects (as shown in Figure 9f) because of the foreground aperture problem [18].

**Figure 12:**
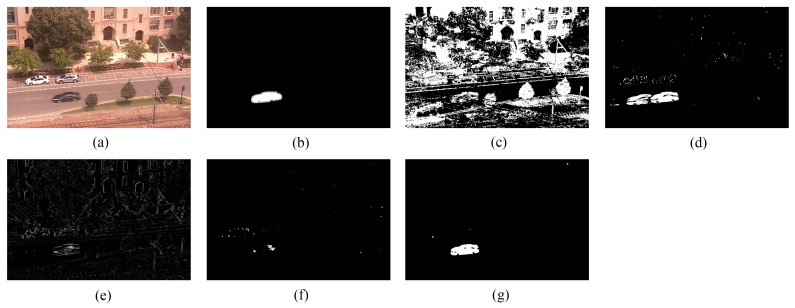
Detection results of Frame 1581 of the intermittent pan sequence: (**a**) input frame; (**b**) ground-truth reference; (**c**) result of ViBe; (**d**) result of PCP; (**e**) result of TD-2DDFT; (**f**) result of TD-2DUWT; (**g**) result of TD-3DDWT.

Figure 12 displays the detection results of Frame 1581 of the intermittent pan sequence. Frame 1581 is the first frame when the PTZ camera stops panning and points to a new field of view, and just from this frame, a moving car transitorily emerges in the PTZ camera’s new field of view until Frame 1587. As can be seen from Figure 12, ViBe detects plenty of false positives, because its background model is not suitable for the new field of view, and there is also no time for the model to adapt to this new field of view. PCP detects a ghost because of its wrong estimation of the background for the new scene. TD-2DDFT yields a result containing edges of the background. In the result of TD-2DUWT, the car is missing due to the foreground aperture problem; whereas our method gains the best result and detects the key target (*i.e.*, the moving car) rapidly and accurately.

**Figure 13:**
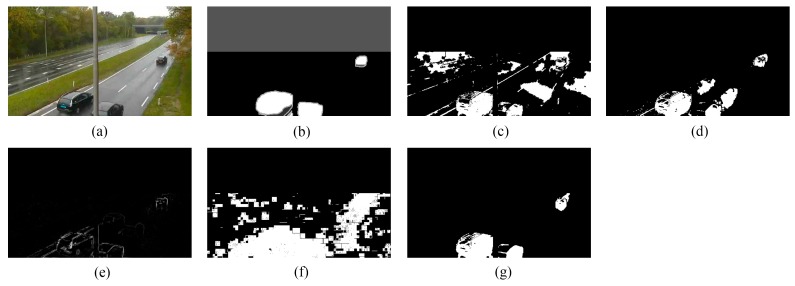
Detection results of Frame 1215 of the two-position pan-tilt-zoom (PTZ) camera sequence: (**a**) input frame; (**b**) ground-truth reference; (**c**) result of ViBe; (**d**) result of PCP; (**e**) result of TD-2DDFT; (**f**) result of TD-2DUWT; (**g**) result of TD-3DDWT.

Figure 13 gives the detection results of Frame 1215 of the two-position PTZ camera sequence. Frame 1215 is the 156th frame when the PTZ camera stops panning and returns to one of its two preset positions. Figure 13 shows that ViBe, PCP and TD-2DUWT all yield many false positives, and TD-2DUWT generates the worst result, because it is sensitive to image noise; whereas our method detects the most accurate and complete foreground objects.

Experimental results demonstrate that our method quickly outputs satisfactory results and outperforms the other algorithms in terms of visual effects. This shows the superiority of our method in dealing with situations without sufficient training time.

### 5.3. Quantitative Comparisons

To assess the detection results objectively, we employ four widely-used metrics (*i.e.*, *Recall*, *Precision*, *F*1 and *Similarity*) to judge the performance of these techniques at the pixel level [23]. Let *TP* be the number of true positives, *TN* the number of true negatives, *FP* the number of false positives, and *FN* the number of false negatives. The four metrics are defined as:

(6) $$Recall=\frac{TP}{TP+FN}$$

(7) $$Precision=\frac{TP}{TP+FP}$$

(8) $$F1=2\frac{Recall\cdot Precision}{Recall+Precision}$$

(9) $$Similarity=\frac{TP}{TP+FN+FP}$$

Since the *Recall* and *Precision* often contradict each other, the overall indicators (*i.e.*, *F*1 and *Similarity*), integrating false positives and false negatives in one single measure, are employed to further compare the results.

The four metrics mentioned above all lie in the range of [0, 1]. The higher the above metrics are, the better the detection results are. As defined in Equations (6)–(9), we need the detected regions to calculate these metrics. However, the detection results of TD-2DDFT only contain the object boundaries, without the object inner parts. Therefore, for a fair comparison, we do not evaluate TD-2DDFT in the quantitative analysis.

#### 5.3.1. Quantitative Comparisons for Situations without Sufficient Training Time

Table 1, Table 2 and Table 3 show the metrics for one typical frame in each indoor short clip, respectively. Clearly, for short clips, ViBe yields unacceptable results with quite low metrics, because its background models need a calibration phase after quick initialization; whereas PCP, TD-2DUWT and TD-3DDWT produce much better results, and TD-3DDWT ranks first with regard to *Precision* for all three indoor short clips. Moreover, in terms of the overall indicators (*i.e.*, *F*1 and *Similarity*), TD-3DDWT performs the best.

Table 4, Table 5 and Table 6 show the average metrics for all 96 frames in each outdoor short clip, respectively. Employing all of the frames of short clips as test data makes our comparison more objective. As shown in these tables, TD-3DDWT ranks first by a large margin concerning the overall indicators. ViBe still produces unsatisfactory results with very low metrics, especially for TC96 (as shown in Table 5); TD-2DUWT suffers from the foreground aperture problem (e.g., Table 4). These results further prove the superiority of our method in analyzing short clips with insufficient training time.

Table 7 and Table 8 exhibit the average metrics for the two PTZ camera sequences, *i.e.*, the intermittent pan sequence and the two-position PTZ camera sequence, respectively. These metrics are calculated utilizing all of the ground-truth references available, that is Frames 1200 to 3500 in the intermittent pan sequence and Frames 800 to 2300 in the two-position PTZ camera sequence, except for the frames captured when the camera is panning.

As can be seen from Table 7, for the intermittent pan sequence, ViBe produces a large amount of false positives due to intermittent panning movements of the PTZ camera. PCP and TD-2DUWT both yield many false negatives, because PCP usually wrongly estimates the backgrounds of new scenes, and TD-2DUWT suffers from the foreground aperture problem. Clearly, for all of the metrics, TD-3DDWT performs the best.

As shown in Table 8, for the two-position PTZ camera sequence, ViBe still cannot adapt its background model to environmental changes in time and, hence, generates plenty of false positives. TD-2DUWT performs the worst, because it is sensitive to image noise and falsely detects a large portion of pixels as foreground pixels (e.g., Figure 13f), which although resulting in a high *Recall*, also results in a surprisingly low *Precision*; whereas TD-3DDWT behaves the best and ranks first by a large margin in terms of the overall indicators (*i.e.*, *F*1 and *Similarity*).

#### 5.3.2. Quantitative Comparisons for Situations without Clean Training Data

Table 9, Table 10 and Table 11 show the average metrics for the three crowded indoor sequences, *i.e.*, the shopping center sequence, the airport sequence and the buffet restaurant sequence, respectively. Because the provided ground-truth references (twenty for each indoor sequence) are not at the start of the test sequences, ViBe has a sufficiently long calibration phase to build accurate background models and performs better than PCP and TD-2DUWT; whereas our method achieves the best results in the crowded indoor scenes, for we remove the disturbance and utilize an adaptive binary detection threshold. Especially, with regard to the overall indicators (*i.e.*, *F*1 and *Similarity*), our method shows its superiority over the other methods.

Table 12, Table 13 and Table 14 show the average metrics for the three crowded outdoor sequences, *i.e.*, the skating sequence, the tram crossroad sequence and the turnpike sequence, respectively. These metrics are calculated utilizing all of the ground-truth references available; that is, Frames 800 to 3900 in the skating sequence; Frames 400 to 900 in the tram crossroad sequence; Frames 800 to 1500 in the turnpike sequence. Despite hundreds of initial frames (without ground-truth references) not being used in the evaluation, our method still performs the best in the crowded outdoor scenes. These results further prove the ability of our method in dealing with crowded scenes without clean training data.

The quantitative comparisons have indicated that our method has a remarkable advantage over other methods in coping with challenging situations lacking training opportunities that occur frequently in real scenes and is of great significance to applications that require rapid and accurate detection of key targets.

#### 5.3.3. Quantitative Comparisons for Normal Videos

Additionally, in order to further testify to the performance of our method in normal conditions, we also provide the category-average metrics for the baseline category of the CDnet in Table 15. The category-average metrics are calculated with no post-processing techniques applied to the methods, with the purpose to evaluate the unaided strength of each method. To calculate the category-average metrics for the baseline category, we utilize all of the ground-truth references available; that is, Frames 470 to 1700 in the highway sequence; Frames 570 to 2050 in the office sequence; Frames 300 to 1099 in the pedestrians sequence; Frames 300 to 1200 in the PETS2006 sequence. For each sequence, hundreds of initial frames without ground-truth references (generally prepared for training those methods that require a training phase) are not used in the evaluation. Under such conditions, our method outperforms PCP and TD-2DUWT and has satisfactory performance comparable to ViBe.

Since our method employs batch processing, we naturally consider it as an offline method. Its memory cost is moderate, for it only stores 32 gray-scale images each time, and no background model is maintained in the memory. Its main computation cost is on the 3D DWT and 3D inverse DWT. In our experiments, the proposed method is implemented on a PC with an i7-2600 3.4-GHz processor and 4 G RAM. Our MATLAB algorithm (including loading the test images and writing the binary results) achieves the processing speeds of 13 fps, 37 fps and 46 fps for the shopping center sequence (with a size of 320×256), the airport sequence (with a size of 176×144) and the buffet restaurant sequence (with a size of 160×120), respectively. Therefore, the computational complexity and memory cost are acceptable in real applications.

## 6. Conclusions

Without a separate training phase, we propose a background subtraction method based on 3D DWT. Considering that static backgrounds correspond to the low-frequency components, we remove the static backgrounds indirectly in the 3D wavelet domain. Additionally, we make use of wavelet shrinkage to remove disturbance and introduce an adaptive threshold based on the entropy of the histogram to obtain optimal detection results. Experimental results demonstrate that our method has a remarkable advantage in coping with situations lacking training opportunities (such as short clips, PTZ camera sequences and long crowded sequences) and outperforms several popular methods. This prominent strength makes our method applicable to many tough situations requiring rapid and accurate detection of key targets.

## Figures and Tables

**Table 1:** Comparison of metrics for the eighth frame of SC96 (Frame 1827).

Method	*Recall*	*Precision*	*F*1	*Similarity*
ViBe	0.5932	0.4992	0.5422	0.3719
PCP	0.6090	0.7541	0.6738	0.5081
TD-2DUWT	0.7262	0.5718	0.6398	0.4704
TD-3DDWT	0.5970	0.8758	0.7103	0.5508

**Table 2:** Comparison of metrics for the 10th frame of AP96 (Frame 3409).

Method	*Recall*	*Precision*	*F*1	*Similarity*
ViBe	0.5377	0.4796	0.5070	0.3396
PCP	0.4678	0.8887	0.6130	0.4419
TD-2DUWT	0.6759	0.6594	0.6676	0.5010
TD-3DDWT	0.5893	0.9248	0.7199	0.5623

**Table 3:** Comparison of metrics for the ninth frame of BR96 (Frame 1558).

Method	*Recall*	*Precision*	*F*1	*Similarity*
ViBe	0.5610	0.3139	0.4026	0.2520
PCP	0.5800	0.7864	0.6676	0.5011
TD-2DUWT	0.5786	0.6474	0.6111	0.4400
TD-3DDWT	0.6278	0.8281	0.7142	0.5554

**Table 4:** Comparison of average metrics for SK96.

Method	*Recall*	*Precision*	*F*1	*Similarity*
ViBe	0.5210	0.3799	0.4394	0.2816
PCP	0.4654	0.5205	0.4914	0.3257
TD-2DUWT	0.2266	0.6287	0.3332	0.1999
TD-3DDWT	0.6120	0.6528	0.6336	0.4637

**Table 5:** Comparison of average metrics for TC96.

Method	*Recall*	*Precision*	*F*1	*Similarity*
ViBe	0.6095	0.0982	0.1692	0.0924
PCP	0.5939	0.8300	0.6924	0.5295
TD-2DUWT	0.5940	0.5574	0.5751	0.4036
TD-3DDWT	0.7502	0.8759	0.8082	0.6781

**Table 6:** Comparison of average metrics for TP96.

Method	*Recall*	*Precision*	*F*1	*Similarity*
ViBe	0.6490	0.2487	0.3596	0.2192
PCP	0.3875	0.9564	0.5515	0.3807
TD-2DUWT	0.7496	0.4212	0.5394	0.3693
TD-3DDWT	0.7234	0.9059	0.8044	0.6728

**Table 7:** Comparison of average metrics for the intermittent pan sequence.

Method	*Recall*	*Precision*	*F*1	*Similarity*
ViBe	0.5047	0.0384	0.0714	0.0370
PCP	0.4227	0.6818	0.5129	0.3405
TD-2DUWT	0.3278	0.7127	0.4491	0.2896
TD-3DDWT	0.6801	0.7363	0.7071	0.5142

**Table 8:** Comparison of average metrics for the two-position PTZ camera sequence.

Method	*Recall*	*Precision*	*F*1	*Similarity*
ViBe	0.6226	0.1052	0.1800	0.0989
PCP	0.6727	0.4325	0.5265	0.3573
TD-2DUWT	0.9461	0.0592	0.1114	0.0590
TD-3DDWT	0.8258	0.7797	0.8021	0.6634

**Table 9:** Comparison of average metrics for the shopping center sequence.

Method	*Recall*	*Precision*	*F*1	*Similarity*
ViBe	0.5981	0.7984	0.6839	0.5196
PCP	0.5509	0.8158	0.6576	0.4899
TD-2DUWT	0.6423	0.5871	0.6135	0.4425
TD-3DDWT	0.5956	0.8726	0.7080	0.5394

**Table 10:** Comparison of average metrics for the airport sequence.

Method	*Recall*	*Precision*	*F*1	*Similarity*
ViBe	0.6233	0.8059	0.7029	0.5419
PCP	0.5383	0.7862	0.6390	0.4836
TD-2DUWT	0.5376	0.5363	0.5369	0.3670
TD-3DDWT	0.5885	0.9075	0.7140	0.5552

**Table 11:** Comparison of average metrics for the buffet restaurant sequence.

Method	*Recall*	*Precision*	*F*1	*Similarity*
ViBe	0.5431	0.7458	0.6285	0.4583
PCP	0.5213	0.7708	0.6220	0.4514
TD-2DUWT	0.5539	0.5410	0.5474	0.3768
TD-3DDWT	0.5612	0.8655	0.6809	0.5162

**Table 12:** Comparison of average metrics for the skating sequence.

Method	*Recall*	*Precision*	*F*1	*Similarity*
ViBe	0.7975	0.7530	0.7746	0.6321
PCP	0.4169	0.4459	0.4309	0.2746
TD-2DUWT	0.1864	0.2795	0.2237	0.1259
TD-3DDWT	0.7559	0.9372	0.8368	0.7195

**Table 13:** Comparison of average metrics for the tram crossroad sequence.

Method	*Recall*	*Precision*	*F*1	*Similarity*
ViBe	0.7195	0.3850	0.5016	0.3347
PCP	0.6193	0.6128	0.6160	0.4451
TD-2DUWT	0.6749	0.4906	0.5682	0.3968
TD-3DDWT	0.7481	0.8446	0.7935	0.6576

**Table 14:** Comparison of average metrics for the turnpike sequence.

Method	*Recall*	*Precision*	*F*1	*Similarity*
ViBe	0.6667	0.8303	0.7396	0.5868
PCP	0.3786	0.9584	0.5429	0.3726
TD-2DUWT	0.8002	0.4066	0.5392	0.3691
TD-3DDWT	0.7440	0.9188	0.8222	0.6981

**Table 15:** Comparison of category-average metrics for the baseline category ^1^.

Method	*Recall*	*Precision*	*F*1	*Similarity*
ViBe	0.7888	0.9046	0.8416	0.7297
PCP	0.5203	0.6913	0.5569	0.4199
TD-2DUWT	0.5438	0.5112	0.4681	0.3274
TD-3DDWT	0.7372	0.9521	0.8279	0.7142

^1^ The category-average metrics are calculated with no post-processing techniques applied to these methods, with the purpose to evaluate the unaided strength of each method.
